# Physiological and Pathological Functions of SLC26A6

**DOI:** 10.3389/fmed.2020.618256

**Published:** 2021-01-21

**Authors:** Juan Wang, Wenkang Wang, Hui Wang, Biguang Tuo

**Affiliations:** ^1^Department of Gastroenterology, Affiliated Hospital of Zunyi Medical University, Zunyi, China; ^2^Department of Critical Care Medicine of the Third Affiliated Hospital (The First People's Hospital of Zunyi City), Zunyi Medical University, Zunyi, China

**Keywords:** SLC26A6, pancreas, intestine, kidney, heart, placenta

## Abstract

Solute Carrier Family 26 (SLC26) is a conserved anion transporter family with 10 members in human (SLC26A1-A11, A10 being a pseudogene). All SLC26 genes except for SLC26A5 (prestin) are versatile anion exchangers with notable ability to transport a variety of anions. SLC26A6 has the most extensive exchange functions in the SLC26 family and is widely expressed in various organs and tissues of mammals. SLC26A6 has some special properties that make it play a particularly important role in ion homeostasis and acid-base balance. In the past few years, the function of SLC26A6 in the diseases has received increasing attention. SLC26A6 not only participates in the development of intestinal and pancreatic diseases but also serves a significant role in mediating nephrolithiasis, fetal skeletal dysplasia and arrhythmia. This review aims to explore the role of SLC26A6 in physiology and pathophysiology of relative mammalian organs to guide in-depth studies about related diseases of human.

## Introduction

Transmembrane transport of anionic substrates is of crucially important for human-body water and electrolyte homeostasis, CO_2_ transport, pH modulation and buffering, absorption of nutrient and vitamin, absorption and secretion of fluid, osmoregulation of cells, neurotransmission, and metabolic processes. SLC4 family and SLC26 family have been reported to mediate the transport of cellular anions, and the SLC26 family serves as a more vital role in mediating the exchange of Cl^−^/${\text{HCO}}_{3}^{-}$ in the epithelial tissues. SLC26 family members are multifunctional transporters which transport monovalent and divalent anions in the body. Among the 10 members of this family, mutations and polymorphisms of several members have been correlated with human diseases (Table 1) (1–7). In addition, pathological phenotypes have been reported in knockout mice deficient in expression of other SLC26 isoforms for which human mutations have not yet been associated with diseases (Table 1) (8–11). As the member which has the most extensive transport functions in the family, SLC26A6 can mediate the transport of Cl^−^/${\text{HCO}}_{3}^{-}$ as well as other anions which include Cl^−^/formate, Cl^−^/oxalate, Cl^−^/nitrate, ${\text{SO}}_{4}^{2-}$/oxalate and Cl^−^/OH^−^ (12, 13). However, in non-epithelial tissues, SLC26A6 functions predominantly as Cl^−^/${\text{HCO}}_{3}^{-}$ and OH^−^ exchangers (14). SLC26A6 is strongly expressed in the intercalated ducts of pancreas (15, 16), proximal tubules of kidney, and proximal small intestine (17, 18). The expression has also been found in the heart (19), reproductive system (20), placenta (21), parotid gland (22), esophagus (23), stomach (24), and even teeth (25, 26) of mammals. Systematic study on the distribution of SLC26A6 in human tissues is lacking, and it may be difficult to detect by Western blot and immunohistochemistry because the members of SLC26 family are heavily glycosylated and SLC26A6 band size varies in the analysis of Western blot (27), especially in other species than mouse, in which comparison with knockout tissue is beneficial. However, there have been reports confirming the expression of SLC26A6 mRNA in human pancreas (28, 29), duodenum (30), kidney (29, 31), and placenta (32). Recently, animal model studies have found that SLC26A6 is crucial significance for the physiology and normal functions of several organs (such as pancreas, intestine, heart, and kidney). However, the mechanism of SLC26A6 exerts in transport process still needs to be elucidated and little is known about the function and dysregulation of SLC26A6 contributes to the manifestations of commonly diagnosed human diseases. This review offers a glimpse into the function of SLC26A6 associated with anions transport and related diseases of mammalian relative organs.

**Table 1:** SLC26A6 deletion related diseases.

**Members**	**Human diseases**	**Members**	**Animal models diseases**
SLC26A2	Chondrodysplasia	SLC26A1^−^/^−^, SLC26A6^−^/^−^	Hyperoxaluria, Nephrolithiasis
SLC26A3	Chloride diarrhea		
SLC26A4	Pendred's syndrome	SLC26A7^−^/^−^, SLC26A9^−^/^−^	Gastric achlorhydria
SLC26A8	Male infertility	SLC26A9^−^/^−^	Distal renal tubular acidosis
SLC26A9	Cystic fibrosis-associated meconium ileus		
	Diabetes		
SLC26A11	Cytotoxic brain edema		

## Structural Features of SLC26A6

**Figure 1:**
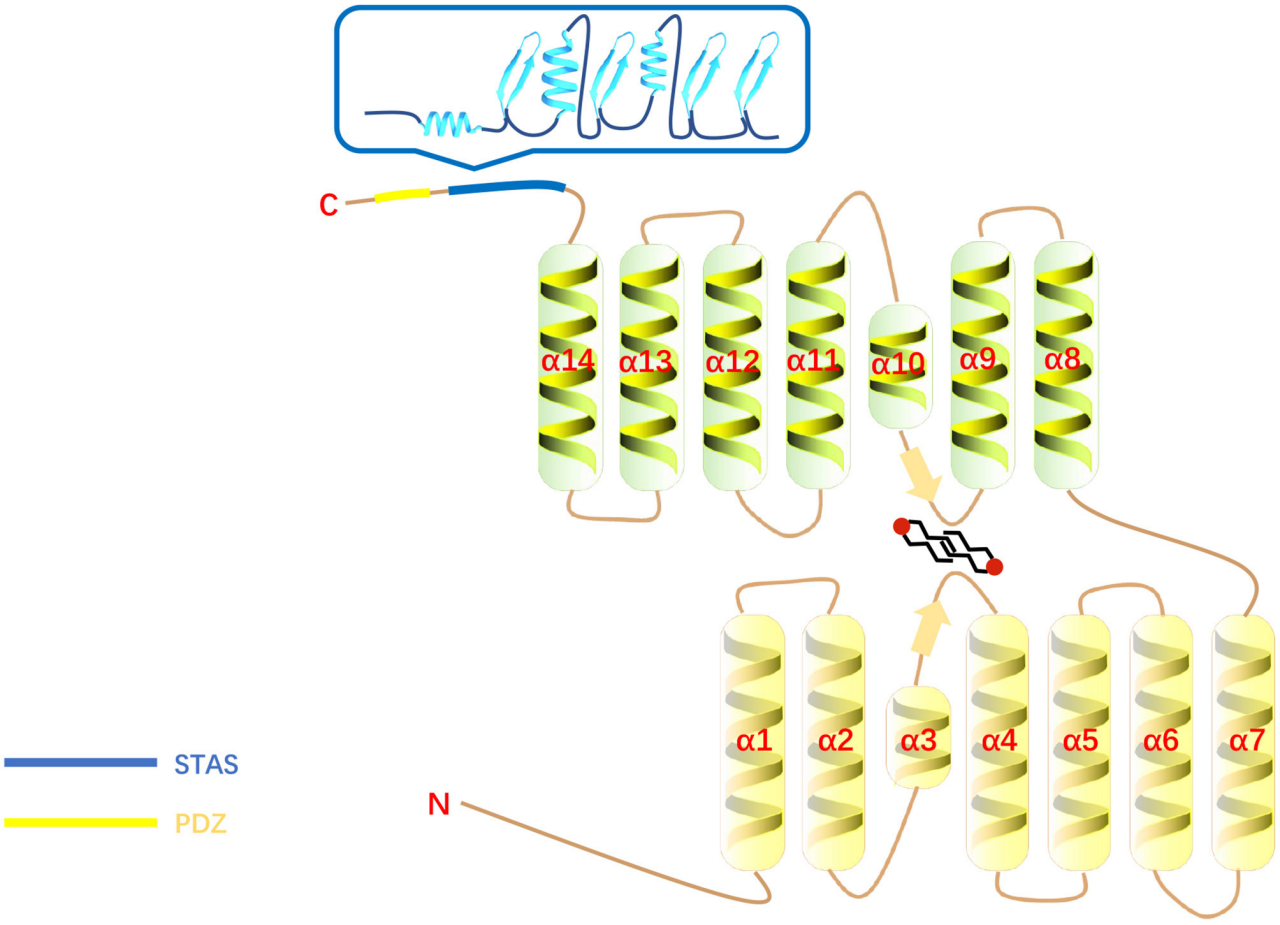
The structure of SLC26A6. The blue box is the STAS domain.

SLC26A6 is a transmembrane secondary transporter (symporters and exchangers) with a molecular mass of 83 kDa and consists of 759 amino acids, which is located on chromosome 3. The membrane-inserted domain of SLC26A6 consists of 14 variable-length α-helices, including two short helices (the 3rd and 10th helices) which do not span the entire width of the lipid bilayer (33). The 14 transmembrane segments are divided into two intertwined inverted repeats parts and 7 transmembrane segments each (33). The core domain contributes a pair of pseudosymmetry-related helices (α-helices 3 and 10) at the top of the cavity that point from opposite directions toward the hydrophobic center of the bilayer (33). There is a STAS (sulfate transporter and anti-sigma factor antagonist) domain at the C-terminus of SLC26A6 (14). The STAS domain is compact and contains a core which consists of just three α-helices and four β-strands (33) and is relevant for intracellular trafficking (34, 35) as well as protein–protein interactions (36, 37). Its deletion impairs substrate transport by the membrane domain (33). In addition, the C-terminus of SLC26A6 contains a consensus PDZ (PSD-95/Disc-large/ZO-1) interaction motif identical to that of the cystic fibrosis transmembrane conductance regulator (CFTR) (38) and the PDZ domains provide places for protein-protein interaction that plays an essential role in the assembly of multiprotein complexes and ultimately involves in regulation of membrane proteins, determining cell polarity, and plasma membrane targeting (39) (Figure 1). There are four isoforms of SLC26A6 have been demonstrated. SLC26A6A and SLC26A6B were cloned from the total RNA of the mouse intestine (13). SLC26A6A is a longer isoform, consisting of 758 amino acids, and SLC26A6B is the shorter isoform with 735 amino acids (28, 29). There are different opinions about which of these two isoforms correspond to the human isoform (28, 29). SLC26A6C lacks 38 amino acids by missing exon 6 and lacks 1 amino acid by using an alternative splice donor and acceptor site at the beginning of exon 17. SLC26A6D had unspliced intron after exon 16 resulting in frame-shift and early termination (38).

## Electrogenic Property of SLC26A6

There is controversy about whether Cl^−^/${\text{HCO}}_{3}^{-}$ exchange mediated by SC26A6 is electrogenic. Initially, Ko et al. detected Cl^−^ and ${\text{HCO}}_{3}^{-}$ transport in Xenopus oocytes expressed SLC26A6 and they demonstrated that the transporter is electrogenic, with unique property (40). Then they expressed SLC26A6 in Xenopus oocytes and HEK293 cells again to confirm that SLC26A6 behaves as an electrogenic transporter based on electrophysiological and pH fluorescence experiments (41). Xie et al. thought that both SLC26A6 and SLC26A3 are electrogenic, but with opposite polarities. SLC26A6 appears to have a Cl^−^:${\text{HCO}}_{3}^{-}$ stoichiometry of 1:2, while the ratio for SLC26A3 is 2:1 (13). In the case of SLC26A3 depletion, the inward current may originate from the SLC26A6. The expression of two members in the same cells (to be precise in the case of epithelia and in the same membrane domain), one with the Cl^−^/${\text{HCO}}_{3}^{-}$ stoichiometry of SLC26A6 and one with that of SLC26A3, results in electroneutral Cl^−^/${\text{HCO}}_{3}^{-}$ exchanges apparently (40). This indicates that the stoichiometry of the two transporting Cl^−^/${\text{HCO}}_{3}^{-}$ is opposite, which may lead to the formation of reverse current. The opposite is that Chernova et al. expressed the homolog of SLC26A6 in Xenopus oocytes and concluded that both human and mouse SLC26A6 mediate the electroneutral Cl^−^/${\text{HCO}}_{3}^{-}$ exchange (42). In addition, SLC26A6-null mouse intestinal transepithelial studies also show that Cl^−^/${\text{HCO}}_{3}^{-}$ exchange by SLC26A6 is electroneutral. Similarly, the current electricity generation and transport stoichiometry of human and mouse SLC26A3 are also controversial and most studies agree that not only human but also mouse SLC26A3 Cl^−^/${\text{HCO}}_{3}^{-}$ exchanges are electroneutral (stoichiometry 1Cl^−^/1${\text{HCO}}_{3}^{-}$) exchange (43–45). Interestingly, Chernova et al. considered that mouse SLC26A6 mediated Cl^−^/oxalate exchange was apparently electrogenic, whereas that mediated by human SLC26A6 appeared electroneutral at the same time (42). However, Cl^−^/oxalate exchange in the apical membrane vesicles of rat mediated by SLC26A6 is also thought to be electrogenic in another study (46). On the contrary, unlike Cl^−^/oxalate exchange, Cl^−^/formate exchange is electroneutral (47).

## SLC26A6 and the Pancreas

### Physiological Role of SLC26A6 in the Pancreas

**Figure 2:**
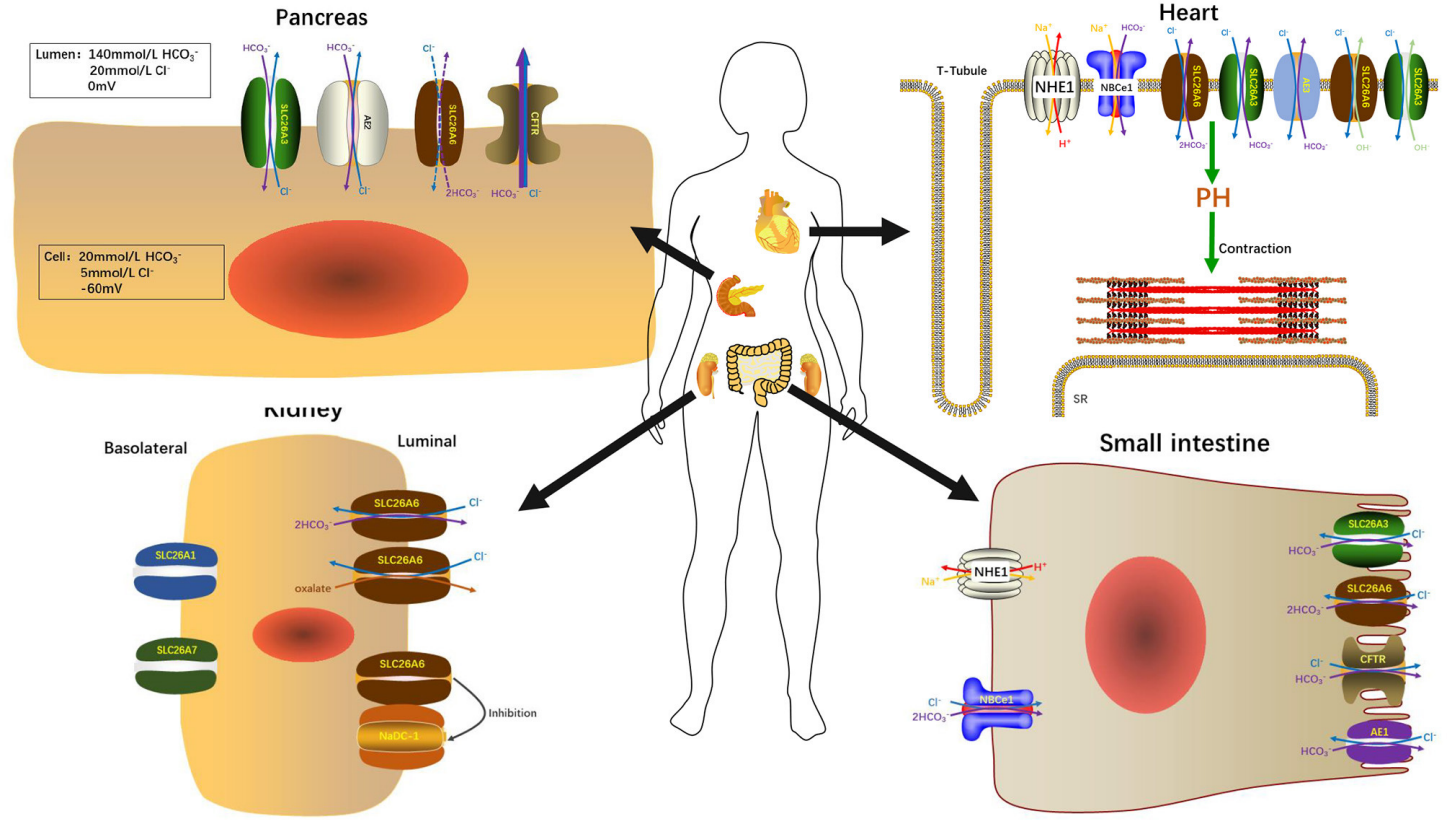
SLC26A6 participates in the maintenance of normal organ function in the pancreas, kidneys, heart, and small intestine.

**Figure 3:**
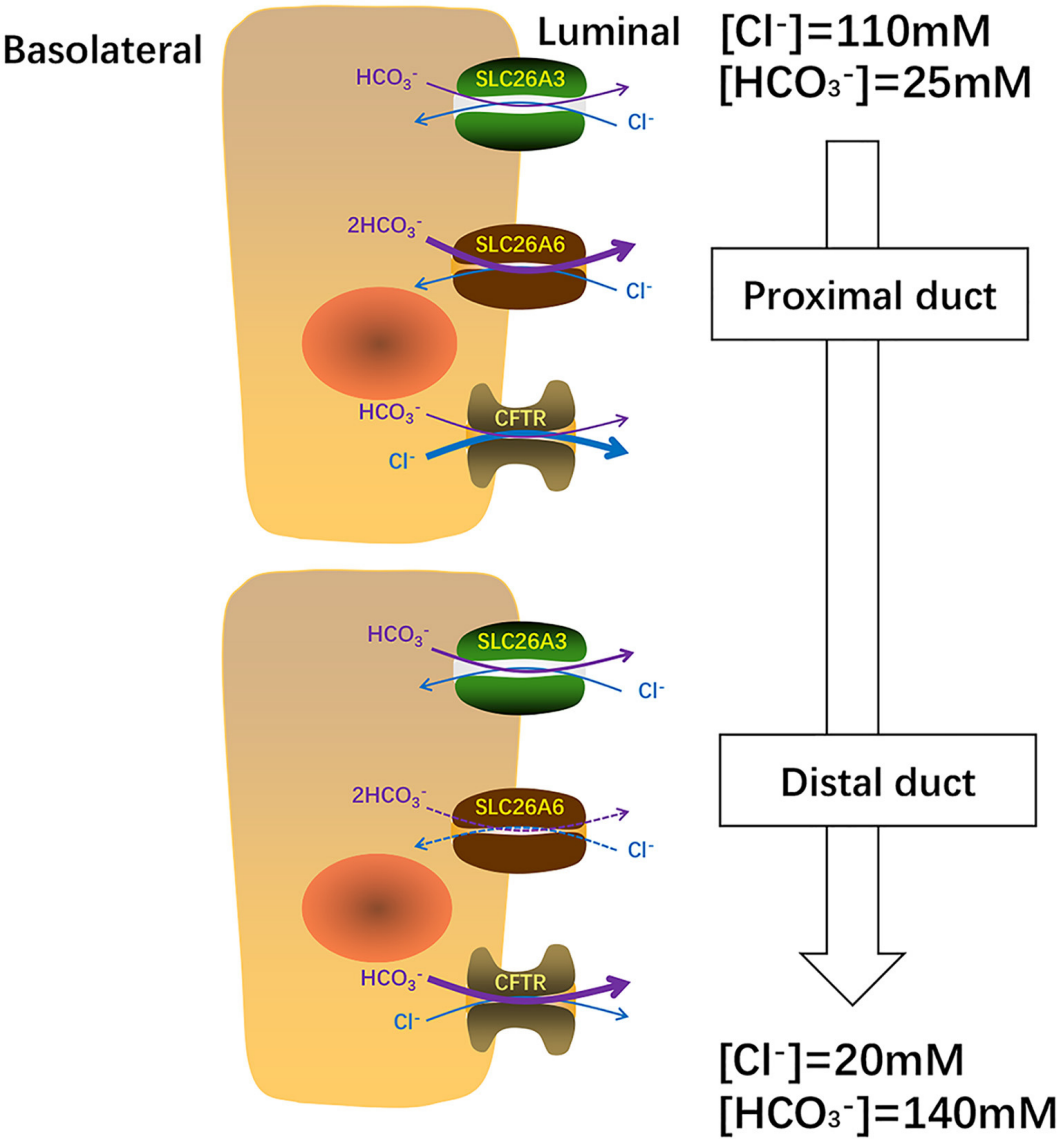
In pancreas, SLC26A6 and SLC26A3 perform their functions at different ion concentrations. SLC26A6 is predominantly responsible for the excretion of basal ${\text{HCO}}_{3}^{-}$ and CFTR is primarily responsible for Cl^−^ secretion from the proximal duct. Close to the distal tubule, the concentration of ${\text{HCO}}_{3}^{-}$ increases, SLC26A3 plays a major role in the secretion of basal ${\text{HCO}}_{3}^{-}$. When the concentration of ${\text{HCO}}_{3}^{-}$ in the distal tubule reaches a maximum of 140 uM, the secretion of ${\text{HCO}}_{3}^{-}$ mainly depends on CFTR.

The secretion of ${\text{HCO}}_{3}^{-}$ and fluid is an essential function of pancreatic ductal epithelium and is critical for maintaining the integrality of the tissue. The pancreas of human secretes 1–2 L isotonic alkaline fluid every day, in which the concentration of bicarbonate may exceed 140 mM under stimulation (48). Under stimulating conditions, ${\text{HCO}}_{3}^{-}$ secretion depends critically upon the activity of CFTR anion channel (49) which is a cAMP-dependent anion channel located on the apical membrane. This ${\text{HCO}}_{3}^{-}$ rich liquid removes digestive enzymes in the duct branches, facilitates solubilization of macromolecular substances, neutralizes the protons secretion of acinar cells, inhabits premature activation of trypsinogen, and neutralizes gastric acid in the duodenum to provide the optimal pH environment for digestive enzymes. Guinea pigs could secrete pancreatic juice containing 140 mM ${\text{HCO}}_{3}^{-}$ under stimulation by secretin or cAMP, which is similar to the concentration of human pancreatic juice ${\text{HCO}}_{3}^{-}$ (50). Interestingly, Steward et al. explored the ${\text{HCO}}_{3}^{-}$ secretion mechanism of pancreatic ducts by isolating the pancreatic interlobular ducts in guinea pigs, and concluded that only one third of ${\text{HCO}}_{3}^{-}$ is secreted via the apical Cl^−^/${\text{HCO}}_{3}^{-}$ exchangers, and the other two thirds by CFTR (50). The Cl^−^/${\text{HCO}}_{3}^{-}$ exchangers of ductal epithelium have three, two are members of the SLC26 family distributed on the apical membrane and one is AE2 of the SLC4 family which distributed on the basolateral membrane (51–53). Interestingly, when the concentration of ${\text{HCO}}_{3}^{-}$ in the lumen reached the highest value (140 mM), Cl^−^/${\text{HCO}}_{3}^{-}$ exchange mediated by SLC26A3 and AE2 would be reversed, which reabsorbs ${\text{HCO}}_{3}^{-}$ instead of secretion (50). The function of SLC26A6 to secrete ${\text{HCO}}_{3}^{-}$ into the lumen is inhibited and the continuing secretion of ${\text{HCO}}_{3}^{-}$ will be mediated almost entirely (90%) by CFTR (49) (Figure 2). Ko et al. demonstrated that binding the highly conserved STAS domains of SLC26A6 to the regulatory (R) domains of CFTR can enhance the activity of both SLC26A6 and CFTR (36). Therefore, SLC26A6 has synergistic interactions with CFTR in the ${\text{HCO}}_{3}^{-}$ secretion of pancreatic ductal epithelium, and SLC26A6 deletion results in dis-regulation of CFTR in the pancreatic duct (54). Most people may think that SLC26A6 deletion could upregulate the ability of CFTR in a compensatory way to secrete ${\text{HCO}}_{3}^{-}$, but there is no evidence to confirm this hypothesis. The explanation given by Wang et al. is that the activity of CFTR is always inhibited by SLC26A6 in the resting state, and SLC26A6 regulates the activity of CFTR by reducing the rate of CFTR activation in the stimulated state at physiological stimulus intensity (54). Therefore, SLC26A6 deletion could remove tonic inhibition of CFTR by SLC26A6 in the resting ducts and by reducing activation of CFTR by SLC26A6 in the stimulated ducts. Conversely, Ishiguro et al. (55) revealed that there was no change of SLC26A6 deletion on neither spontaneous nor stimulated secretion in isolated ducts or *in vivo*. However, reverse transcription (RT)-PCR showed an apparent upregulation of SLC26A3 mRNA, which may be a compensatory upregulation of SLC26A6 deletion to maintain bicarbonate secretion and pancreatic juice volume. Since SLC26A6 is predominantly distributed in the proximal portion of pancreas duct and SLC26A3 is distributed in the distal portion (36, 40), they are activated by different ion gradients (Figure 3).

### SLC26A6 and Pancreatic Diseases

The distribution and role of SLC26A6 in human pancreatic ducts is still unclear. The pathological significance of SLC26A6 in the pancreas is little known. Deterioration of Pancreatic ductal ${\text{HCO}}_{3}^{-}$ secretion is observed in chronic pancreatitis. Therefore, SLC26A6 is a reasonable candidate for a chronic pancreatitis susceptibility gene. But the study from Balazs et al. verified the SLC26A6 is not related to mutations associated with chronic pancreatitis (56). Unfortunately, there is no more study on human pancreatic diseases. Further studies on the role of the anion exchanger in the human pancreas may clarify the role of ${\text{HCO}}_{3}^{-}$ secretion disorders in acute pancreatitis, chronic pancreatitis and related pancreatic diseases.

## SLC26A6 and the Intestine

### Physiologic Role of SLC26A6 in the Intestine

#### SLC26A6 and Intestinal Cl^–^/${\text{HCO}}_{3}^{\text{–}}$ Exchange

**Figure 4:**
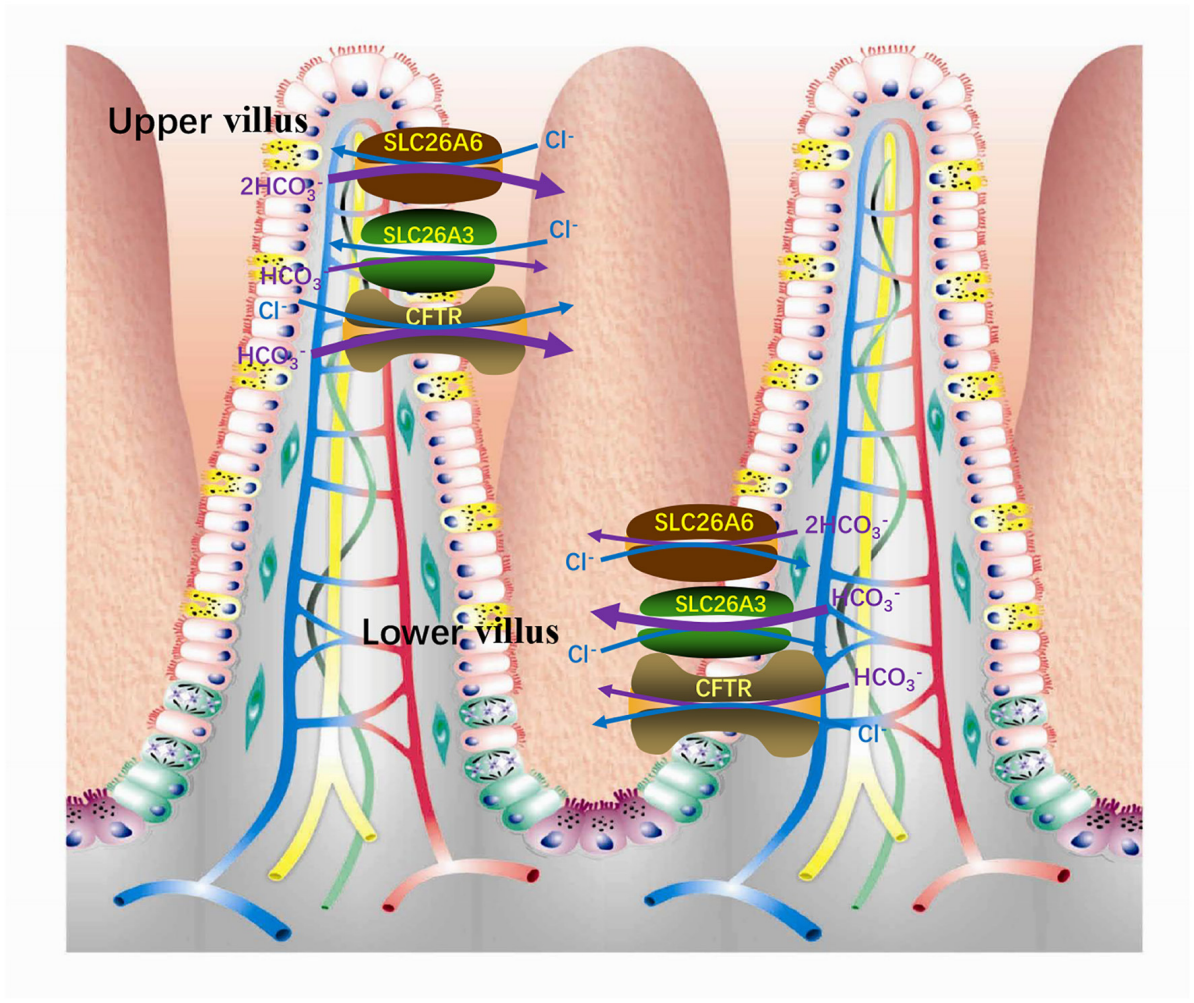
In the small intestinal villi epithelium, SLC26A6 is predominantly responsible for the basal ${\text{HCO}}_{3}^{-}$ secretion of the upper villi, while SLC26A3 is responsible for the lower villi.

It is known that intestinal ${\text{HCO}}_{3}^{-}$ secretion is stimulated by cAMP (57–59). There are at least two secretion mechanisms of ${\text{HCO}}_{3}^{-}$, one is Cl^−^-dependent and the other is Cl^−^-independent. The apical membrane Cl^−^-independent ${\text{HCO}}_{3}^{-}$ secretion stimulated by cAMP is considered to be mediated by the Cl^−^ channel. CFTR is one of the Cl^−^ channels responsible for ${\text{HCO}}_{3}^{-}$ secretion (60–62). Under stimulation, CFTR is still the most important conduction pathway for intestinal secretion of ${\text{HCO}}_{3}^{-}$ (63). On the country, the apical membrane Cl^−^-dependent ${\text{HCO}}_{3}^{-}$ secretion is mediated by the Cl^−^/${\text{HCO}}_{3}^{-}$ exchangers (60, 62). SLC26A6 and SLC26A3 are considered to play vital roles in Cl^−^-dependent ${\text{HCO}}_{3}^{-}$ secretion, primarily responsible for basal ${\text{HCO}}_{3}^{-}$ secretion related strongly to blood ${\text{HCO}}_{3}^{-}$ concentration (64). In addition, there are three mechanisms involved in intestinal acid-base transport (Figure 3), a DIDS-sensitive Na^+^/${\text{HCO}}_{3}^{-}$ co-transporter (NBC) and an amiloride-sensitive Na^+^/H^+^ exchanger (NHE1) present on the basolateral membranes and a Cl^−^ /${\text{HCO}}_{3}^{-}$ (AE4) exchanger located on the apical membranes of entrocyte (60, 65, 66). SLC26A3 and SLC26A6 are both expressed in the small intestine and large intestine. The difference is that SLC26A6 expression is very high in the small intestine (especially in the duodenum) but low in the colon (17) and SLC26A3 is expressed primarily in the colon and moderately in the small intestine (44). Therefore, SLC26A6 is particularly vital in mediating the increase of duodenal ${\text{HCO}}_{3}^{-}$ secretion with the raising blood ${\text{HCO}}_{3}^{-}$ (64). Also, *in vitro* studies have shown that its presence enhances the secretory rate of ${\text{HCO}}_{3}^{-}$ in the basal state (18, 67). The role of SLC26A6 in duodenal ${\text{HCO}}_{3}^{-}$ secretion crucially depends on the acid/base status under *in vivo* conditions, with very crucial contribution to basal secretory rates at high, but not at low, blood ${\text{HCO}}_{3}^{-}$ concentrations (64). In addition, SLC26A6 mediates ${\text{HCO}}_{3}^{-}$ secretion strongly dependent on carbonic anhydrase, while the Cl^−^-independent and cAMP-activated ${\text{HCO}}_{3}^{-}$ secretion are both largely independent of the enzyme (68). Based on SLC26A6-null mice, it was found that SLC26A6 is responsible for <30% of the basal ${\text{HCO}}_{3}^{-}$ secretion and had no respondence to cAMP and forskolin simulation, while prostaglandin E_2_-stimulated ${\text{HCO}}_{3}^{-}$ secretion is mediated via a Ca^2+^-dependent pathway (18, 67). The SLC26A3-mediated Cl^−^/${\text{HCO}}_{3}^{-}$ exchange contributes to the rest of ~60% of basal ${\text{HCO}}_{3}^{-}$ secretion (69). The mouse small intestine was further measured which revealed that the level of SLC26A6 and SLC26A3 expression reciprocates along the villus axis with SLC26A6 greatest in the upper villus in the lower villus/crypt and SLC26A3 greatest in the lower villus/crypt (Figure 4), but both exchangers are well-represented throughout the villus length (69, 70). And microfluorimetry studies of the upper villus epithelium found that SLC26A6 provides 70% of the total Cl^−^/${\text{HCO}}_{3}^{-}$ exchange, while SLC26A3 provides nearly all of the apical membrane Cl^−^/${\text{HCO}}_{3}^{-}$ exchange of the lower villus epithelium (71). SLC26A6 has been demonstrated to also participate in the transport of Cl^−^/${\text{HCO}}_{3}^{-}$ in the lower villi, but since SLC26A6 is an electrogenic transporter, cell acidification or membrane depolarization during glucose transport could mask the activity of SLC26A6 (72). Moreover, SLC26A6 may primarily serve other significant functions, such as the absorption of salt, nutrient-associated anion absorption (73) and regulating the pH of the intestinal lumen fluid. Studies demonstrated that compared with wild-type, SLC26A6-null mice show a decreased basal duodenal ${\text{HCO}}_{3}^{-}$ secretory rate (18). But the effects of SLC26A6-null mice on intestine and tubular functions are not profound at the steady state (18). This does not rule out that SLC26A6 deletion may lead to the compensatory upregulation of other Cl^−^-absorption transporters in the intestinal lumen to maintain homeostasis.

#### SLC26A6 and Intestinal Cl^–^/Oxalate Exchange

**Figure 5:**
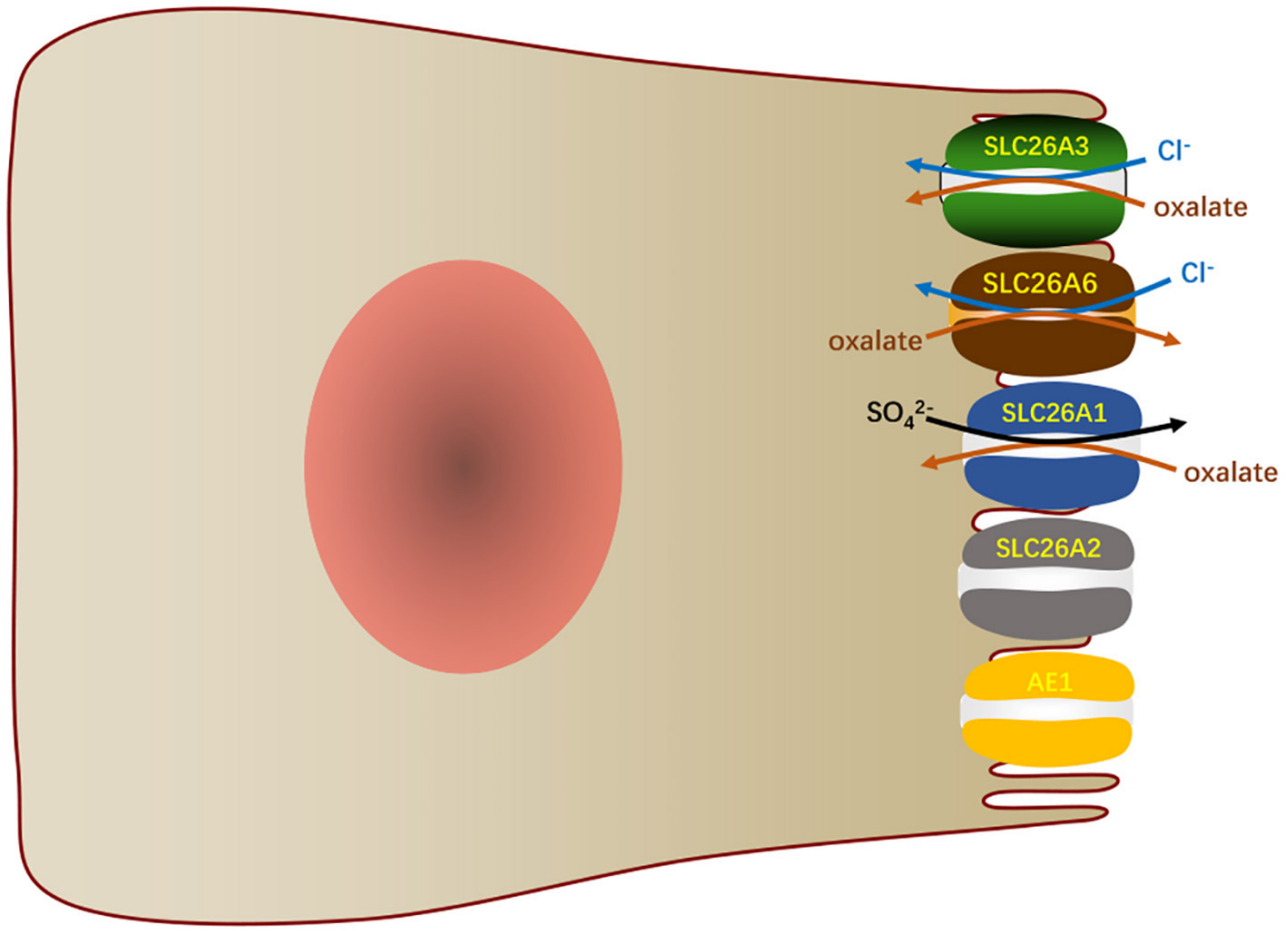
In the small intestinal villi epithelium, SLC26A6 and other transports participate in the oxalate transport.

Another function of SLC26A6 in the small intestine is to mediate oxalate excretion by the duodenum (9) and distal ileum (74), which plays an important role in oxalate homeostasis. Oxalate is indeed an obdurate anion and exists as a monovalent or divalent anion. It is not metabolized in the human body, but it is easily produced through enormous metabolic pathways (75). It is usually present in minute amounts related to other anions, yet small changes in its concentration in the existence of calcium can result in the deposition of calcium oxalate. The concentration of oxalate in plasma depends on dietary load, intestinal absorption, metabolic production and renal excretion. The transport of oxalate in intestine is bidirectional and net transport (76, 77). Transport methods include transcellular pathway and paracellular pathway. Studies demonstrated that intestinal oxalate secretion relies on a SLC26A6-dependent transcellular mechanism, while the oxalate absorption takes place by a paracellular channel (78). In general, in contrast to this phenomenon of basal net oxalate secretion in the small bowel and proximal colons, the distal colons of animals typically exhibit basal net absorption of oxalate (79, 80). SLC26A6 in the mouse ileum mediates apical secretion of oxalate in exchange for Cl^−^ and is an important component of the transcellular serosal-to-mucosal unidirectional oxalate flux (74). The vectorial oxalate transport mediated by SLC26A6 appears to be more dependent on the direction and magnitude of counterion driver gradients than the intrinsic property of the protein (81). Although SLC26A6 is also expressed in the apical membranes of mouse colon (17), its role in colonic oxalate transport awaits further studies. In addition, SLC26A1, SLC26A2, and SLC26A3 also participate in the transport of intestinal oxalate (Figure 5). SLC26A1 is considered to a ${\text{SO}}_{4}^{2-}$/oxalate transporter. It may mediate ${\text{SO}}_{4}^{2-}$/oxalate exchange in parallel with the Cl^−^/oxalate exchange, again leading to the recycling of oxalate with minimal net oxalate transport (8, 74). SLC26A2 participates in the secretion of intestinal oxalate with trans-${\text{SO}}_{4}^{2-}$, Cl^−^, or oxalate itself (82). SLC26A3 mediates oxalate transport by absorbing oxalate and Cl^−^ in the large and small intestines (83). AE1 is also detected to express in the apical membranes of ileum (84) and surface cells of the distal colon (85), which may also play a role in oxalate transport.

### SLC26A6 and Intestinal Diseases

Duodenal mucosal ${\text{HCO}}_{3}^{-}$ secretion plays important role in protecting duodenal mucosa against gastric-induced injure (86, 87). Clinical study found that duodenal mucosal ${\text{HCO}}_{3}^{-}$ secretion was found to be markedly diminished in Helicobacter pylori-associated duodenal ulcer patients (61). *Helicobacter pylori* infection impaired the expressions and functional activities of duodenal mucosal CFTR and SLC26A6 via TGFβ-mediated P38 MAPK signaling pathway, which contributed to the development of duodenal ulcer (88, 89). The studies showed that inflammatory bowel disease (IBD) patients usually accompanied with hyperoxaluria and kidney stones (90, 91), which might be due to the lack of oxalate transporters. Also, hyperoxaluria is a major complication after malabsorptive bariatric surgery of obesity (92, 93) and more than 50% of patients would be complicated. Previous studies demonstrated that SLC26A6-null mice have serious defects in the intestinal secretion of oxalate which could lead to enhanced net oxalate absorption and result in a high incidence of hyperoxemia, hyperuricemia and calcium oxalate urolithiasis (9, 74). And nephrolithiasis in the SLC26A6-null mouse is accompanied by 50–75% reduction in intestinal oxalate secretion with increased intestinal oxalate absorption (82). It has been shown that the activation of PKA signaling pathway can enhance SLC26A6 surface protein expression and increase the intrinsic activity of preexisting SLC26A6 surface membrane transporters to stimulate oxalate transport (94). Therefore, SLC26A6 may be used as one of the therapeutic targets for hyperoxaluria caused by various reasons.

## SLC26A6 and the Kidney

### Physiological Role of SLC26A6 in the Kidney

There are two ways for the body to excrete oxalate, one is via the intestinal tract as mentioned above, and the other is by the kidneys. The majority of oxalate excretion in the human body though the kidneys where 90–95% of circulating oxalate is removed via the urine (95, 96), and the rest is secreted into the intestine. When intestinal or kidney oxalate metabolism is defective, it can lead to hyperoxaluria which is the main risk factor for kidney stone formation (97). Calcium oxalate is the primary element in 70–80% of kidney stones (98), and the risk of stone formation was also affected by small changes in urinary oxalate concentration (99). The expression of SLC26A6 can be detected on the apical membrane of the proximal duct epithelium of the kidney (12), and the pivotal function of the proximal tubule is to retrieve the majority of NaCl and water filtered by the kidney. Previous studies have shown that oxalate cloud stimulate the reabsorption of Cl^−^ in the proximal tubule, which means that the apical membrane Cl^−^/oxalate exchange mediates Cl^−^ absorption as well as oxalate secretion (100–102). Moreover, microperfusion studies found that SLC26A6 was also the major apical Cl^−^/${\text{HCO}}_{3}^{-}$ exchangers in the proximal tubule straight segments. Wang et al. proposed that the NaCl reabsorption mediated by the Cl^−^/oxalate exchange in the proximal tubule was entirely contributed by SLC26A6, and the apical membrane Cl^−^/${\text{HCO}}_{3}^{-}$ exchange mediated by SLC26A6 was not a contributing factor to transtubular NaCl absorption (18). The Cl^−^/oxalate exchange mediated by SLC26A6 in the proximal tubule is essential for oxalate homeostasis (74). In addition, AE1 and SLC26A7 distributed on the basolateral membrane might participate in the transport of oxalate in the tubule epithelium (46, 103). SLC26A1 is considered to mediate basolateral oxalate-${\text{SO}}_{4}^{2-}$/${\text{HCO}}_{3}^{-}$ exchange in the proximal tubule (46). The function of SLC26A7 in the transport of oxalate is unclear. In addition to mediate oxalate secretion, SLC26A6 also can form a complex with the succinate transporter NaDC-1 and strongly inhibit NaDC-1 activity and interact with NaDC-1 to control absorption of citrate from the urinary lumen (104). Urinary citrate can chelate free Ca^2+^ to protect against Ca^2+^ oxalate crystallization. However, whether this mechanism can prevent the occurrence of kidney stones remains to be further studied.

### SLC26A6 and Renal Diseases

Studies have indicated that SLC26A6-null mice have a 4-fold increase in urine oxalate excretion (9, 74), but serum oxalate levels were not apparently different between KO and WT mice, although there was a tendency toward hyperoxalemia in the KO mice (74). The high concentration of oxalate in the urine makes the kidney stones appear high frequency in SLC26A6-null mice, and the occurrence rate of male mice is higher than that of female mice (9). In SLC26A6-null mice, histological examination of the kidney demonstrated that the stones mainly comprise of calcium oxalate, and are primarily found in the lumen of cortical tubules and in the urinary space (9). Although a number of studies have shown a close relationship between the expression of SLC26A6 and kidney stone formation, but its precise role in the human diseases remain unknown (104–107). Studies have demonstrated that SLC26A6 has fundamental roles not only in proximal tubule NaCl transport but also in the prevention of hyperoxaluria as well as calcium oxalate nephrolithiasis. Therefore, further studies to explore the metabolism of oxalate, absorption and excretion in clinic is vital importance, and it may enhance oxalate excretion in pharmacology to provide new therapeutic targets.

## SLC26A6 and the Heart

### Physiological Role of SLC26A6 in the Heart

The homeostasis of cardiomyocytes depends on cytoplasmic buffer and membrane ion transporter, thus the regulation of pH in cardiomyocytes is extremely complicated. pH is a vital regulator of cardiac excitation and contraction (108), which is an adverse contributing factor of electric arrhythmia and cardiac hypertrophy (109). An uncompensated decrease in cytoplasmic pH leads to abnormal electrical activities, reducing Ca^2+^ transient and contraction in cardiomyocytes by potentially decreasing the binding of Ca^2+^ to troponin C as well as by affecting cross-bridges action resulting in maximal force reduction (108, 110), which would trigger arrhythmia (111). Inversely, twitch tension, resting tonic tension, voltage dependent tonic tension, and after-contraction contractile parameters can be enhanced with intracellular alkalosis in sheep cardiac fibers (112). H^+^ equivalent transporters Na^+^/H^+^ exchanger (NHE) and Na^+^-${\text{HCO}}_{3}^{-}$ co-transporter (NBC) mediate acid extrusion, while Cl^−^/${\text{HCO}}_{3}^{-}$ exchangers and Cl^−^/OH^−^ exchangers extrude excess base (113, 114). Also, sarcolemmal lactic acid transporter is recruited in response to increased anaerobic metabolism (115). Under normal physiological state, the regulating system can maintain the pH at a steady state value of 7.2. When coupled with a Na^+^-dependent acid excretion mechanism, Na^+^ loading increased Cl^−^/${\text{HCO}}_{3}^{-}$ exchange which can affect myocardial contractility and promote cardiac hypertrophy (116). Another function of Cl^−^/${\text{HCO}}_{3}^{-}$ exchange in the heart is to counter the alkalinizing effects of Na^+^/H^+^ exchange (116, 117), which can reduce the occurrence of cardiac hypertrophy (118, 119). SLC26A6, SLC26A3, and AEs (especially AE3) have been found to mediate Cl^−^/${\text{HCO}}_{3}^{-}$ exchange in the myocardium (19, 120) (Figure 3). Immunohistochemistry showed that SLC26A6, SLC26A3, and AE3 exist in the plasma membrane of ventricular myocytes (19). The expression level of SLC26A6 is lower in the atrium than in the ventricle, while AE3 is only detected in the ventricle (19). SLC26A6 is the major anion exchanger in the heart because the heart-dependent acid load is primarily mediated by SLC26A6 and its expression in the myocardial cell membrane is much higher than that of AE and SLC26A3 (19, 121). Furthermore, recovery from alkalinization induced by acetate is severely impaired in SLC26A6-null mice cardiomyocytes (121). Of note that SLC26A6 is a dual Cl^−^/${\text{HCO}}_{3}^{-}$, Cl^−^/OH^−^ exchanger with unique implications for myocardial intracellular pH regulation (19). Also, SLC26A6 is the most important Cl^−^/${\text{HCO}}_{3}^{-}$ and Cl^−^/OH^−^ exchanger for the myocardium maintaining normal activity. However, SLC26A6 mediates the physiological relevance of the Cl^−^/OH^−^ exchange in the heart is not clear yet. In addition, SLC26A6 and SLC26A3 are the only Cl^−^/${\text{HCO}}_{3}^{-}$ exchangers of the SLC26 family that are expressed in the heart (19). The cardiac SLC26A6 not only regulates the pH, but also plays a unique role in regulating cardiac excitability and function of heart. The study of Sirish et al. demonstrated that SLC26A6 deletion resulted in the shortening of cardiac action potential (AP), cardiomyocyte Ca^2+^ transient and sarcoplasmic reticulum Ca^2+^ loading decrease, cardiomyocyte diminution of sarcomeric shortening, and cardiomyocyte pH elevation (121). In SLC26A6-null mice, these factors lead to a decrease of cardiac fractional shortening and cardiac contractility responses and alter cardiac conduction system, as seen in sinus bradycardia and fragmentation of the QRS electrocardiographic-recorded complex (121).

### SLC26A6 and Heart Diseases

SLC26A6 is considered to be a primary transporter in the heart ventricle. Although the regulation mechanism of various ion transporters is complicated (19), studies have demonstrated that the expression of SLC26A6 and Cl^−^ transporting activity are upregulated in the type 2 diabetic heart model, which reveals that effective SLC26A6 blockers may be efficient in regulating pH of type 2 diabetic hearts (122). Additionally, Cl^−^ influx and ${\text{HCO}}_{3}^{-}$ efflux mediated by SLC26A6 may be beneficial to intracellular acidification in diabetic myocardium during cardioplegia-induced arrest (122). Evidences reveal that protein kinase C (PKC) can inhibit the transport activity of SLC26A6 (19, 123). The inhibitory effect of PKC is attributed to PKC-mediated displacement owing to the combination of carbonic anhydrase II and SLC26A6, and thus destroys the ${\text{HCO}}_{3}^{-}$ transport metabolites (123). Therefore, in order to prevent myocardial hypertrophy, attention should be paid to regulate the function of myocardial SLC26A6 to ensure the acid-base homeostasis of myocardial tissue. In the state of anesthesia and intensive care, patients with basic heart diseases should minimize the use of vasoactive drugs that stimulate α1-adrenergic receptors to avoid insufficient myocardial contraction.

## SLC26A6 and the Placenta

Sulfate is significant for human growth and development and human usually get it from metabolism of sulfur-containing amino acids and the diet in the body. Also, sulfate is an essential nutrient for the growth and development of fetus (124). However, the developing fetus have negligible capacity to generate sulfate from methionine and cysteine (125, 126) and depends on sulfate supplied from maternal circulation via placental sulfate transporters (127). For human, several ${\text{SO}}_{4}^{2-}$ transporters have been detected in the placenta, which include SLC26A6 (21). During the period of pregnancy, the level of maternal circulating sulfate increases by nearly 2-fold (128), and the increased plasma sulfate levels are associated with elevated sulfate reabsorption of kidney (129). In the kidney, sulfate is filtered in the glomerulus and then reabsorbed via epithelial cells of the proximal tubule, firstly across the apical membrane where SLC13A1, SLC26A2, and SLC26A6 are expressed and secondly via SLC26A1 on the basolateral membrane (31, 46, 130). Therefore, maintaining the normal function of ${\text{SO}}_{4}^{2-}$ transporters, such as SLC26A6 in the placenta is essential for fetal growth and development. In future clinical applications, monitoring the function of placental ${\text{SO}}_{4}^{2-}$ transporters may be used to predict and evaluate neonatal cartilage development.

## Conclusion

In summary, SLC26A6 transporter mediates the exchange of anions in mammalian cells, thereby participating in the maintenance of normal physiological functions of various organs. However, there are still many problems on SLC26A6 need to be solved. Firstly, the transport base of SLC26A6 has not yet been illuminated at the molecular level. Secondly, the cellular mechanism that regulates and fine-tune the activity of SLC26A6 transporter has not been elucidated. Finally, it is about the influence of the normal function and dysfunction of SLC26A6 on human related diseases. The connection and role of SLC26A6 with the body may become a research hotspot and may become a new molecular marker for the diagnosis and treatment of human-related diseases. The development of drugs targeting SLC26A6 will provide new treatment directions for human-related diseases.

## Author Contributions

JW and WW wrote the manuscript. HW participated in information collection, analysis, and organization. BT revised the manuscript for clarity and style. All authors contributed to the article and approved the submitted version.

## Conflict of Interest

The authors declare that the research was conducted in the absence of any commercial or financial relationships that could be construed as a potential conflict of interest.
